# Monkeys and Humans Share a Common Computation for Face/Voice Integration

**DOI:** 10.1371/journal.pcbi.1002165

**Published:** 2011-09-29

**Authors:** Chandramouli Chandrasekaran, Luis Lemus, Andrea Trubanova, Matthias Gondan, Asif A. Ghazanfar

**Affiliations:** 1 Neuroscience Institute, Princeton University, Princeton, New Jersey, United States of America; 2 Department of Psychology, Princeton University, Princeton, New Jersey, United States of America; 3 Marcus Autism Center, Emory University School of Medicine, Atlanta, Georgia, United States of America; 4 Department of Psychology, University of Regensburg, Regensburg, Germany; 5 Medical Biometry and Informatics, University of Heidelberg, Heidelberg, Germany; 6 Department of Ecology and Evolutionary Biology, Princeton University, Princeton, New Jersey, United States of America; Indiana University, United States of America

## Abstract

Speech production involves the movement of the mouth and other regions of the face resulting in visual motion cues. These visual cues enhance intelligibility and detection of auditory speech. As such, face-to-face speech is fundamentally a multisensory phenomenon. If speech is fundamentally multisensory, it should be reflected in the evolution of vocal communication: similar behavioral effects should be observed in other primates. Old World monkeys share with humans vocal production biomechanics and communicate face-to-face with vocalizations. It is unknown, however, if they, too, combine faces and voices to enhance their perception of vocalizations. We show that they do: monkeys combine faces and voices in noisy environments to enhance their detection of vocalizations. Their behavior parallels that of humans performing an identical task. We explored what common computational mechanism(s) could explain the pattern of results we observed across species. Standard explanations or models such as the principle of inverse effectiveness and a “race” model failed to account for their behavior patterns. Conversely, a “superposition model”, positing the linear summation of activity patterns in response to visual and auditory components of vocalizations, served as a straightforward but powerful explanatory mechanism for the observed behaviors in both species. As such, it represents a putative homologous mechanism for integrating faces and voices across primates.

## Author Summary

The evolution of speech is one of our most fascinating and enduring mysteries—enduring partly because all the critical features of speech (brains, vocal tracts, ancestral speech-like sounds) do not fossilize. Furthermore, it is becoming increasingly clear that speech is, by default, a multimodal phenomenon: we use both faces and voices together to communicate. Thus, understanding the evolution of speech requires a comparative approach using closely-related extant primate species and recognition that vocal communication is audiovisual. Using computer-generated avatar faces, we compared the integration of faces and voices in monkeys and humans performing an identical detection task. Both species responded faster when faces and voices were presented together relative to the face or voice alone. While the details sometimes appeared to differ, the behavior of both species could be well explained by a “superposition” model positing the linear summation of activity patterns in response to visual and auditory components of vocalizations. Other, more popular computational models of multisensory integration failed to explain our data. Thus, the superposition model represents a putative homologous mechanism for integrating faces and voices across primate species.

## Introduction

When we speak, our face moves and deforms the mouth and other regions [1], [2], [3], [4], [5]. These dynamics and deformations lead to a variety of visual motion cues (“visual speech”) related to the auditory components of speech and are integral to face-to-face communication. In noisy, real world environments, visual speech can provide considerable intelligibility benefits to the perception of auditory speech [6], [7], faster reaction times [8], [9], and is hard to ignore—integrating readily and automatically with auditory speech [10]. For these and other reasons, it's been argued that audiovisual (or “multisensory”) speech is the primary mode of speech perception and is not a capacity that is simply piggy-backed onto auditory speech perception [11].

If the processing of multisensory signals forms the default mode of speech perception, then this should be reflected in the evolution of vocal communication. Naturally, any vertebrate organism (from fishes and frogs, to birds and dogs) that produces vocalizations will have a simple, concomitant visual motion in the area of the mouth. However, in the primate lineage, both the number and diversity of muscles innervating the face [12], [13], [14] and the amount of neural control related to facial movement [15], [16], [17], [18] increased over time relative to other taxa. This ultimately allowed the production of a greater diversity of facial and vocal expressions in primates [19], with different patterns of facial motion uniquely linked to different vocal expressions [20], [21]. This is similar to what is observed in humans. In macaque monkeys, for example, coo calls, like the /u/ in speech, are produced with the lips protruded, while screams, like the /i/ in speech, are produced with the lips retracted [20].

These and other homologies between human and nonhuman primate vocal production [22] imply that the mechanisms underlying multisensory vocal *perception* should also be homologous across primate species. Three lines of evidence suggest that perceptual mechanisms may be shared as well. First, nonhuman primates, like human infants [23], [24], [25], can match facial expressions to their appropriate vocal expressions [26], [27], [28], [29]. Second, monkeys also use eye movement strategies similar to human strategies when viewing dynamic, vocalizing faces [30], [31], [32]. The third, indirect line of evidence comes from neurophysiological work. Regions of the neocortex that are modulated by audiovisual speech in humans [e.g., 8,33,34,35,36,37], such as the superior temporal sulcus, prefrontal cortex and auditory cortex, are similarly modulated by species-specific audiovisual communication signals in the macaque monkey [38], [39], [40], [41], [42], [43]. However, none of these behavioral and neurophysiological results from nonhuman primates provide evidence for the critical feature of human audiovisual speech: a *behavioral advantage* via integration of the two signal components of speech (faces and voices) over either component alone. Henceforth, we define “integration” as a statistically significant difference between the responses to audiovisual versus auditory-only and visual-only conditions[44].

For a homologous perceptual mechanism to evolve in monkeys, apes and humans from a common ancestor, there must be some behavioral advantage to justify devoting the neural resources mediating such a mechanism. One behavioral advantage conferred by audiovisual speech in humans is faster detection of speech sounds in noisy environments—faster than if only the auditory or visual component is available [8], [9], [45], [46]. Here, in a task operationalizing the perception of natural audiovisual communication signals in noisy environments, we tested macaque monkeys on an audiovisual ‘coo call’ detection task using computer-generated monkey avatars. We then compared their performance with that of humans performing an identical task, where the only difference was that humans detected /u/ sounds made by human avatars. Behavioral patterns in response to audiovisual, visual and auditory vocalizations were used to test if any of the classical principles or mechanisms of multisensory integration [e.g. 47,48,49,50,51,52,53] could serve as homologous computational mechanism(s) mediating the perception of audiovisual communication signals.

We report two main findings. First, monkeys integrate faces and voices. They exhibit faster reaction times to faces and voices presented together relative to faces or voices presented alone —and this behavior closely parallels the behavior of humans in the same task. Second, after testing multiple computational mechanisms for multisensory integration, we found that a simple superposition model, which posits the linear summation of activity from visual and auditory channels, is a likely homologous mechanism. This model explains both the monkey and human behavioral patterns.

## Materials and Methods

### Ethics statement

All experiments and surgical procedures were performed in compliance with the guidelines of the Princeton University Institutional Animal Care and Use Committee. For human participants, all procedures were approved by the Institutional Review Board at Princeton University. Informed consent was obtained from all human participants.

### Subjects

Nonhuman primate subjects were two adult male macaques *(Macaca fascicularis*). These monkeys were born in captivity and provided various sources of enrichment, including cartoons displayed on a large screen TV as well as olfactory, auditory and visual contact with conspecifics. The monkeys underwent sterile surgery for the implantation of a head-post.

The human participants consisted of staff or graduate students (n = 6, 4 males, mean age  = 27) at Princeton University. Two of the subjects were authors on the paper (CC, LL). The other four human subjects were naïve to the purposes and goals of the experiment.

### Avatars

We would like to briefly explain here why we chose to use avatars. First, it is quite difficult to record monkey vocalizations which only contain mouth motion without other dynamic motion components such as arbitrary head motion and rotation— which themselves may lead to audiovisual integration [54]. Second, start and end positions of the head from such videos of vocalizations, at least for monkeys, tend to be very variable which would add additional visual motion cues. Third, we wanted constant lighting and background and the ability to modulate the size of the mouth opening and thereby parameterize visual stimuli. Fourth, the goal of this experiment was to understand how mouth motion integrated with the auditory components of vocalizations and we wanted to avoid transient visual stimuli. Real videos would not have allowed us to control for these factors; avatars provide us with considerable control.

### Monkey behavior

Experiments were conducted in a sound attenuating radio frequency (RF) enclosure. The monkey sat in a primate chair fixed 74 cm opposite a 19 inch CRT color monitor with a 1280×1024 screen resolution and 75 Hz refresh rate. The 1280×1024 screen subtended a visual angle of ∼25° horizontally and 20° vertically. All stimuli were centrally located on the screen and occupied a total area (including blank regions) of 640×653 pixels. For every session, the monkeys were placed in a restraint chair and head-posted. A depressible lever (ENV-610M, Med Associates) was located at the center-front of the chair. Both monkeys spontaneously used their left hand for responses. Stimulus presentation and data collection were performed using Presentation (Neurobehavioral Systems).

#### Stimuli: Monkeys

We used coo calls from two macaques as the auditory components of vocalizations; these were from individuals that were unknown to the monkey subjects. The auditory vocalizations were resized to a constant duration of 400 milliseconds using a Matlab implementation of a phase vocoder [55] and normalized in amplitude. The visual components of the vocalizations were 400 ms long videos of synthetic monkey agents making a coo vocalization. The animated stimuli were generated using 3D Studio Max 8 (Autodesk) and Poser Pro (Smith Micro), and were extensively modified from a stock model made available by DAZ Productions (Silver key 3D monkey). As a direct stare or eye contact in monkeys means a challenge or a threat, the direction of the gaze of monkey avatars was averted slightly to a target approximately 20 degrees to the left of straight ahead. To increase the realism of the monkey avatars, we used the base skin texture extracted from photographs of real macaques. When presented on the screen, the monkey avatar was 6.5” wide (12.25°) at the shoulder and 4.5” (8.55°) tall from the top of the head to the bottom of the screen. The face itself was 2.75” wide (5.25°) between the eyes and 3” (5.72°) tall from the top of the head to the bottom of the chin, with the width of the face tapering as it neared the mouth. The audiovisual stimuli were generated by presenting both visual and auditory-only components with an 85-millisecond lag between the onset of mouth opening and the sound of the vocalization. Such a lag is within the natural range for macaque monkeys [40].

#### Task structure for monkeys

Monkeys were trained to detect two coo vocalizations according to a redundant target free-response paradigm [56]; detection was indicated by a lever-press. Redundant target paradigms refer to experimental designs where two or more targets appear simultaneously and responses to any target are considered as hits (see for example, [51], [57], [58], [59]). They could be from different modalities (visual and auditory) or from the same modality (color and shape). In our case, the redundant targets were motion of the mouth and the sound of the coo vocalization. Free response paradigms refer to the absence of explicit trial markers [56], [60]. We chose a free response paradigm because it mimics natural audiovisual communication—faces are usually continuously visible and move during vocal production. Coo vocalizations were presented at different loudness levels (50–85 dB) and at random intervals in ∼63 dB spectrally pink background noise; each vocalization was paired with a synthetic monkey face whose mouth opened in a manner concordant with the loudness of the vocalizations. During every block of the experimental session, a face was always visible, but, only moved for its corresponding identity matched vocalization. The identity of the avatar was counter-balanced across blocks of 60 trials with an inter-block interval ranging from 10–12 seconds in duration. The stimuli-- auditory-only, visual-only and audiovisual conditions--were presented at an inter-sound-interval drawn from a uniform distribution between 1 and 3 seconds. Monkeys were trained to respond to the coo vocalization events in visual, auditory or audiovisual conditions while withholding responses when no stimuli were presented. A press of the lever within a window of 135 to 2000 milliseconds after onset of the vocalization event led to a juice reward. Lever presses outside this window were defined as false alarms and a timeout ranging anywhere from 3 to 5.5 seconds was imposed. Any press during this timeout period led to a renewal of the timeout with the duration again randomly drawn from a uniform distribution from 3 to 5.5 seconds. The monkeys had to wait the entire duration of this timeout period before a new stimulus was presented. A session usually lasted from 300 to 600 trials spanning durations of 25 to 50 minutes.

#### Reaction times and accuracy

Reaction times (RT) were measured as the first depression of the lever after onset of the stimulus. In a free response paradigm, one can define hits, misses and false alarms. A response within the 135 – 2000-millisecond window after the onset of the stimulus was rewarded with a drop of juice and defined as a hit. An omitted response in this window was classified as a miss [56], [60]. A response outside this 2 second period where no vocalizations was defined as a false alarm. Hit rate was defined as the ratio of hits to hits plus misses. For each SNR and condition, the accuracy was defined as the ratio of hits to hits plus misses expressed as a percentage. The false alarm rate was defined as number of false alarms divided by the sum of hits and false alarms. We only took sessions where false alarm percentages were low (in the 10 – 20 %) range keeping with prior standards in the literature [61]. Monkeys were trained until false alarms were around 10–20 %. Hit rate and False alarm rate from two example sessions from monkey 1 are shown in Figures S1A, B along with the quantification of the average false alarm rate across sessions (Figure S1C).

#### Training

Monkeys were shaped through standard operant conditioning techniques over several months (8 months for monkey 1 and 5 months for monkey 2) to respond to vocalization events but withhold responses during the inter-stimulus interval. Training was performed by first shaping the monkey to press a lever for juice reward, then to a sound with a large signal to noise and a large window for gaining a reward. Once the monkey had learned to respond reliably to vocalizations (>80 % hit rate) but withheld responses during the inter-stimulus interval (<20 % false alarms), different SNRs were introduced along with a concomitant restriction of the response window to two seconds. Static and moving faces were then introduced and auditory and audiovisual stimuli were randomly presented. Finally, the control condition of visual-only, that is, facial motion without any accompanying vocalization was introduced, leading monkeys to respond reliably to visual-only, auditory-only and audiovisual coo vocalizations.

### Human behavior

Experiments were conducted in a psychophysics booth. The human sat in a comfortable chair approximately 65 cm opposite a 17 inch LCD color monitor with a 1280×1024 screen resolution and 75 Hz refresh rate. The 1280×1024 screen subtended a visual angle of 28 degrees horizontally and 24 degrees vertically. All stimuli were centrally located on the screen and occupied an area of 640×653 pixels. All stimulus presentation and data collection were performed using Presentation (Neurobehavioral Systems).

#### Stimuli: humans

For humans, vocalization stimuli were /u/sounds made by two female undergraduates at Princeton University recorded using a Canon Vixia HD100 digital camcorder. These vocalizations were resized to a constant length of 400 milliseconds and normalized in amplitude. For visual stimuli, we again used 400 ms synthetic avatars created using the Poser Pro program. In particular, we modified the stock poser model (Sydney, Smith micro pro) and used the animation available for making an /u/ sound. Two avatars were created by altering the shape of the face and the hair to generate two different avatars. The human avatar was 5” wide (11.95°) and 6” tall from the top of the head (14 degrees). The face itself was 5” wide (11.95°) between the eyes and 5.5” (13°) tall from the top of the head to the bottom of the chin, with the width of the face decreasing in width near the mouth. Each avatar was paired with a vocalization and this identity correspondence was always maintained.

#### Task structure for humans

For humans, we used an almost identical task structure as the monkeys with minor modifications. In particular, we reduced the timeout periods for false alarms as humans very rarely made them. Second, blocks were longer with number of trials ranging from 90 to 105 trials per block. Each session contained 4 blocks. Again, the order of avatars was randomized and counterbalanced across avatars. Each human subject completed at least 2 sessions, leading to approximately 60 trials per modality and SNR level. Humans pressed the spacebar button on the keyboard to denote successful detection. We measured accuracy and RTs as described above for monkeys.

### Statistical analysis of behavioral performance

#### Comparison between audiovisual, auditory-only and visual-only RTs

We used non-parametric tests for comparing the RT distributions for visual, auditory and audiovisual vocalizations. For single subjects (both monkeys and humans), RTs were compared first by using a Kruskal-Wallis non-parametric ANOVA comparing whether RT distributions were significantly different between visual-only, auditory-only and audiovisual conditions. Post-hoc tests were conducted using Mann-Whitney tests comparing whether these distributions were different on a single subject and SNR basis. For the monkeys, we chose the Mann-Whitney test over paired t-tests because the RT distributions were not normal and also had different number of trials for different conditions (because of misses, etc). In addition, for humans, we performed, a paired samples t-test comparing normalized RTs for the audiovisual to the auditory-only and visual-only conditions.

#### Standard error for means and medians

We computed bootstrap error bars by resampling the raw RT distributions with replacement 1000 times and estimating the standard deviation of the mean of the resampled data.

#### Bootstrap test for benefits in monkeys

To test if values of benefits for monkeys were significantly different from 0, we calculated the difference between mean RT of the audiovisual and the minimum of the mean RTs of the auditory-only and visual-only conditions. We then computed a sampling distribution for these benefits by resampling from the audiovisual and the condition of interest. The difference of the means was considered statistically significantly if the 95% bootstrap confidence interval did not include zero.

#### Deriving a window of integration using pooled data from monkeys and humans

We used the session-by-session variability of monkeys and variance across humans to derive a window of integration. For example, Monkey 1's RTs on three successive days for the loudest SNR of 22 dB were as follows. Day1: V – 598 ms, A – 526 ms, AV – 470. Day 2: V – 779 ms, A – 498 ms, AV – 492 – ms. Day 3: V – 611 ms, A – 492 ms, AV – 438 ms. The benefits, in each of these cases are, 55 ms, 5 ms and 53 ms. The corresponding differences between the visual-only and auditory-only RTs are 71 ms, 281 ms and 118 ms. Thus, when the discrepancy between visual and auditory-only RT was too large (>250 ms) then the benefit was at most 5 ms. On the other hand, when the visual-only and auditory-only RTs were closer (∼80 to 120 ms) then the benefit was robust and was 55 ms in magnitude.

### Race model

Our audiovisual detection experiment is an extension of the classical redundant signals paradigm. In such experiments, it is common to observe that RTs to multisensory targets presented simultaneously are faster than unisensory RTs. This effect is usually termed the “redundant signals effect”. One important class of explanations for the redundant signals effect is the “race model”. According to the race model (or a “parallel first terminating” model), redundancy benefits are not due to an actual integration of visual and auditory cues. To illustrate, assume that the time to detect and respond to a single target—e.g., the facial motion--varies according to a statistical distribution. Similarly, the time to detect and respond to the auditory-only vocalization also varies according to a statistical distribution. Whenever, both facial motion and the vocalization are presented together, the stimulus that is processed faster in a given trial determines the response time. As the RT distributions typically overlap with one another, slow processing times are removed. Thus, RTs to redundant stimuli are always faster than those for the single stimuli. A standard way to test whether this principle can explain RT data is to use the race model inequality [57], which posits that the cumulative RT distribution for the redundant stimuli never exceeds the sum of the RT distributions for the unisensory stimuli. That is, if *F_AV_* (t), *F_V_* (t) and *F_A_* (t) are the estimated cumulative distributions (CDF) of the RTs for the three different modalities
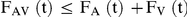
then one cannot rule out race models as an explanation for the facilitation of RT. On the other hand, if this inequality is violated in a given data set, then parallel processing cannot completely account for the benefits observed for multisensory stimuli and an explanation based on co-activation is necessary. We computed the CDFs of our conditions and then computed the difference between the actual CDF of the audiovisual condition and the CDF predicted by the race model. The maximum positive point of this difference was taken as the index of violation, *R*. Positive values of *R* means that the race model is rejected. If this value is 0, then the race model is upheld.

#### Tests of race model violations for single subjects

We needed to test whether these race model violations were statistically significant. To test the violations of the race model on a single subject basis, we compared the true value of R to one computed by a bootstrap method that performs artificial iterations of our multisensory experiments. A variant of this conservative bootstrap method using the area instead of the maximum was originally outlined in seminal studies of behavioral multisensory integration in humans [*51*]. The entire experiment (i.e. AV, V, A) was simulated 10,000 times for each monkey and each SNR with the following steps carried out for each simulation. Simulated RTs for the auditory-only and visual-only conditions were obtained by randomly sampling (with replacement) from the observed distributions of auditory and visual-only RTs for that subject. For the audiovisual condition, in accordance with the race model, the simulated RT was obtained by sampling two RTs (one from the visual RT distribution, one from the auditory RT distribution), and then selecting the minimum of the two values. In addition, as the maximum value for the race model inequality is obtained when the times for the two racers are strongly negatively correlated with one another, we adopted a criterion that introduces a strong negative correlation in the simulation. This was accomplished by randomly selecting an RT from one distribution (e.g., visual-only) that is at a percentile P in the distribution and then sampling the auditory distribution with percentile 100 – P. Thus fast responses to visual motion are paired with slow responses to auditory vocalizations and vice versa, providing a strong and conservative test of the race model. After sampling the appropriate number of trials for each condition, the CDFs and the index of violation (R_b_) for each experiment was obtained. The distribution of R_b_ was then compared to the real value of R. A p-value as a test of significance was obtained by computing the number of simulated values (R_b_) that exceeded the true value R estimated from the data.

#### Tests of race model violations for humans

When data from multiple subjects are available (as in the case of our human data), instead of using bootstrap models, one can test the race model by obtaining the cumulative distribution functions for all subjects and evaluating the consistency of violations of the race model across subjects. To achieve this, we again adapted a permutation test recently developed for testing the violations of the race model. Here we briefly describe the procedure; further details are provided in [*62*]. We first estimated for each participant, the cumulative distribution function for each SNR for auditory, visual and audiovisual conditions, we then computed the difference d for each participant i as
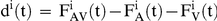
where *F* denotes the estimate of the true cumulative distribution function. When *d^i^* is positive it means that the race model is violated and when d^i^ is negative it means that the pattern of RTs are not different from those predicted by a race model. If *d^i^* follows an approximate normal distribution and *s_d_* is the standard deviation estimated from the sample data, one can then use a one sample t-test to test if this is significant at a single *t*. However, a more robust way that controls for type I error for multiple time points is to use a permutation test. The assumption for this permutation test is as follows. If the race model holds, then the sign of d will be random across participants with some participants showing violations of the race model (*d* is positive) and some showing no violation of the race model (*d* is negative). The average across participants would then be equal to 0. We therefore used a permutation test where we randomly multiplied the participant-specific value of *d* by +1 or -1 (with probability 0.5) and then calculated the t values for multiple time points (280 – 350 ms, into 8 bins). The test statistic for multiple time points then corresponds to the maximum of the t values (so-called t_max_ statistic). We repeated this permutation 10001 times to generate a distribution of the test statistic and computed a p value by identifying the proportion of permuted test statistics that exceeded the true value of the test statistic.

### Superposition models of audiovisual integration

Several models of audiovisual integration have been proposed over the years, but superposition models are simple and possess considerable explanatory power. Here we briefly describe the model, and detailed explanations are available elsewhere [63], [64]. We first consider the case of single modality trials (visual or auditory). We assume that the onset of the stimulus (i.e. visual mouth motion or the auditory vocalization) induces a neural renewal counting process (for examples, action potentials or spikes, but it could be any event which is counted) that counts up to a critical number of events, *c*. The assumption is that, as soon as a critical number of events, *c*, have been registered at some decision mechanism, a response execution process, *M*, which consumes an amount of time with a mean *µ_M_*_,_ is started. The main postulate of the superposition model is that in the audiovisual condition the renewal processes generated by either the visual and the auditory signals are superposed, thereby reducing the waiting time for the critical count. Specifically, if *N_V_* (t) and *N_A_* (t) are themselves counting processes for the visual-only and auditory-only conditions, and the two stimuli are presented simultaneously, that is with 0 lag, then the new process for the audiovisual stimulus is given as
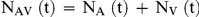



It is immediately apparent that this audiovisual process will reach the critical level of *c* counts faster than the individual auditory and visual processes. If the auditory-only and visual-only stimuli are presented with a lag of say τ, as in our case with visual mouth motion preceding the auditory vocalization by τ milliseconds, then the process becomes,
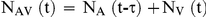



To specify this model fully and test and fit to data, one must specify an inter-arrival distribution. Usually this is assumed to be exponential in nature that leads to a homogenous Poisson counting process. For τ  =  0, the waiting time for the *c^th^* event is well defined and follows a gamma distribution with mean *c*/λ and variance *c*/λ^2^, where λ (λ>0) is the intensity parameter of the Poisson process. For example, the auditory and visual-only RT would then be
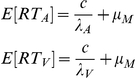



The mean audiovisual RT would be given by the following simple expression. It is the waiting time for the *c* ^th^ with the visual and auditory rates summed and is given as follows.
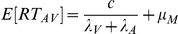



When this model is to be applied when there are differences in the SOAs, that is, τ>0, the waiting time for the *c* ^th^ event is no longer gamma distributed and instead follows a more complicated distribution. Fortunately, this model has been completely explicated and published expressions are already available [63], [64]. The audiovisual RT in this case is the expected value of the waiting time to the *c* ^th^ count.

#### Prediction of audiovisual RTs

We assumed that across all conditions and intensities, the values of c and µ_M_ are constant across conditions. These assumptions are reasonable for the following reasons. A constant c means the criterion is constant across conditions and intensities. A constant value of µ_M_ means that the average motor time is constant across all these conditions. For the five auditory-only and visual-only conditions, we estimated for each of the different SNRs, using the real values of RTs, RT_A_, RT_V_, the values of the rate parameters λ_A_ and λ_V_ for each of the different intensities then are given by the following expressions.
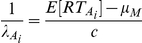


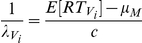

*i* denotes the *i^t^* ^h^ SNR or mouth opening size. We then estimated the audiovisual RTs according to the equation for RT_AV_, we allowed the value of *c* to vary from (2 to 50) and µ_M_ from (150 to 450) and found the best values of *c* and µ_M_ which minimized the least square error between the true audiovisual R and the predicted value of the RT according to the equation. We chose these values of *c* and µ_M_ and then created simulated audiovisual RTs.

#### Simulation of auditory, visual and audiovisual reaction times

To derive simulated auditory, visual and audiovisual RTs and benefit curves, we first found the relationship between λ_Ai_ and SNR at each of the different measured SNRs and then interpolated to get smooth estimates of λ_A_ and SNR. We performed a similar analysis to get an estimate of λ_V_ and values of *c* and *M* were derived from the true data. We then again estimated the value of the audiovisual RT and derived the benefit values in a similar way as the real data.

## Results

**Figure 1:**
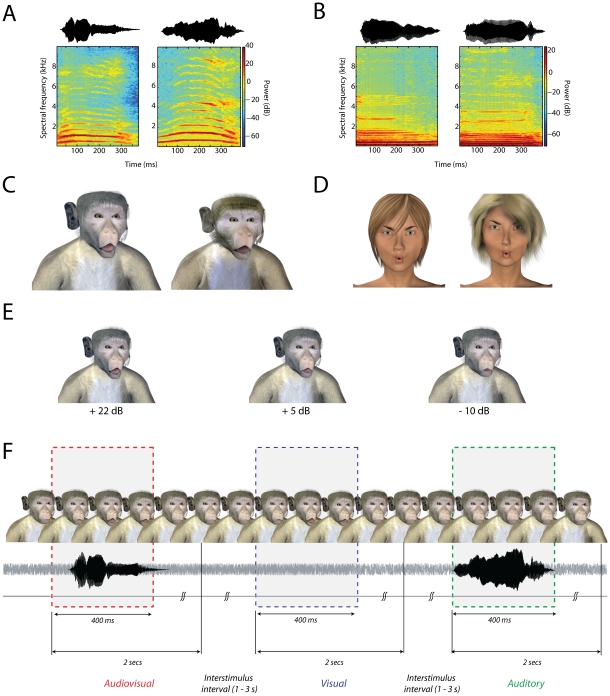
Stimuli and Task structure for monkeys and humans. **A:** Waveform and spectrogram of coo vocalizations detected by the monkeys. **B:** Waveform and spectrogram of the /u/ sound detected by human observers. **C:** Frames of the two monkey avatars at the point of maximal mouth opening for the largest SNR. **D:** Frames of the two human avatars at the point of maximal mouth opening for the largest SNR. **E**: Frames with maximal mouth opening from one of the monkey avatars for three different SNRs of + 22 dB, +5 dB and – 10 dB. **F:** Task structure for monkeys. An avatar face was always on the screen. Visual, auditory and audiovisual stimuli were randomly presented with an inter stimulus interval of 1–3 seconds drawn from a uniform distribution. Responses within a 2 second window after stimulus onset were considered to be hits. Responses in the inter-stimulus interval are considered to be false alarms and led to timeouts.

Monkeys were trained, and humans were asked, to detect auditory, visual or audiovisual vocalizations embedded in noise as fast and as accurately as possible. This task was similar to a redundant signals paradigm [57], and was designed to approximate a natural face-to-face vocal communication event in close encounters. In such settings, the vocal components of the communication signals are degraded by environmental noise. The face and its motion, on the other hand, are usually perceived clearly. In the task, monkeys responded to ‘coo’ calls that are affiliative vocalizations commonly produced by macaque monkeys in a variety of contexts [65,66, Figure 1A]; humans were asked to detect the acoustically similar vowel sound /u/ (Figure 1B). All vocalizations had five different levels of sound intensity and were embedded in a constant background noise. The signal-to-noise ratio (SNR) ranged from −10 dB to +22 dB relative to a background noise of 63 dB. For dynamic faces, we used computer-generated monkey and human avatars (Figures 1C, D). The use of avatars allowed us to restrict facial motion to the mouth region, ensure constant lighting and background, and to parameterize the size of the mouth opening while keeping eye and head positions constant. The degree of mouth-opening was in accordance with the intensity of the associated vocalization: greater sound intensity was coupled to larger mouth openings by the dynamic face (Figure 1E). Two coos and two /u/ sounds were paired with two monkey and human avatars, respectively, and this pairing was kept constant. Furthermore, species-stimuli pairings were kept constant: monkeys only saw and heard monkey vocalizations, and humans only saw and heard human vocalizations. Previous psychophysical and fMRI experiments have successfully used computer-generated human avatars to probe the processing of audiovisual speech [54], [67], [68], [69].

During the task, one avatar face would be continuously on the screen for a block of trials (n = 60); the background noise was also continuous (Figure 1F). In the “visual only (V)” condition, this avatar would move its mouth without any corresponding auditory component; that is, it silently produced a coo for monkey avatars or an /u/for human avatars. In the “auditory-only (A)” condition, the vocalization normally paired with the *other* avatar (which is not on the screen) is presented with the *static* face of the avatar. Finally, in the “audiovisual (AV)” condition, the avatar moves its mouth accompanied by the corresponding vocalization and in accordance (degree of mouth opening) with its intensity. Each condition (V, A, or AV) was presented after a variable interval (drawn from a uniform distribution) between 1 and 3 seconds. Subjects indicated the detection of an event (visible mouth motion, auditory signal or both) by pressing a lever (monkeys) or a key (humans) within 2 seconds following the onset of the stimulus. Every 60 trials, a brief pause was imposed, followed by a new block in which the avatar face was switched on the screen, and the identity of the coo or /u/ sound used for the auditory-only condition was switched as well.

### Accuracy and reaction time

**Figure 2:**
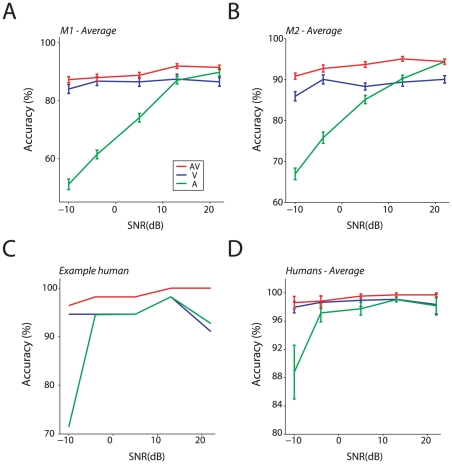
Detection accuracy for monkeys and humans. **A**: Average accuracy across all sessions (n = 48) for Monkey 1 as a function of the SNR for the unisensory and multisensory conditions. Error bars denote standard error of mean across sessions. X-axes denote SNR in dB. Y-axes denote accuracy in %. **B**: Average accuracy across all sessions (n = 48) for Monkey 2 as a function of the SNR for the unisensory and multisensory conditions. Conventions as in A. **C**: Accuracy as a function of the SNR for the unisensory and multisensory conditions from a single human subject. Conventions as in A. **D**: Average accuracy across all human subjects (n = 6) as a function of the SNR for the unisensory and multisensory conditions. Conventions as in A.

We measured the accuracy of the monkeys and humans detecting vocalizations in the audiovisual, auditory-only and visual-only conditions. Figure 2A shows the detection performance of Monkey 1 averaged over all sessions (both coo calls) as a function of SNR for the three conditions of interest. In the case of the visual-only condition, the size of mouth opening has a constant relationship with the auditory SNR and it is thus shown on the same x-axis. In the auditory-only condition, as the coo call intensity increased relative to the background noise, the detection accuracy of the monkey improved. In contrast, modulating the size of the mouth opening in the visual-only condition had only a weak effect on the detection accuracy. Finally, the detection accuracy for audiovisual vocalizations was mildly enhanced relative to the visual-only condition and with very little modulation as a function of the SNR. The same pattern was seen for Monkey 2 (Figure 2B). When the data was pooled over all the SNRs, accuracy was significantly better for both monkeys in the audiovisual condition compared to either unisensory condition (paired t-tests, **Monkey 1**: AV vs V, t (47)  = 3.77, p<.001, AV vs A, t (47)  = 19.94, p<.001; **Monkey 2**: AV vs V, t (47)  = 15.85, p<.001, AV vs A, t (47)  = 8.1, p<.001).

This general pattern was replicated in humans (n = 6). Figure 2C shows the performance of a single human observer on this same task detecting the /u/ sound. Excluding the lowest SNR value in the auditory-only condition, accuracy is almost at ceiling for all three stimulus conditions. The average accuracy over the 6 human subjects as a function of SNR is shown in Figure 2D. Performance pooled across all SNRs was maximal for the audiovisual condition and was enhanced relative to the auditory-only condition (t (5)  = 2.71, p = 0.04). It was not significantly enhanced relative to the visual-only condition (t (5) = 0.97, p = 0.37). The lack of enhancement relative to the visual-only condition is likely because the visual-only performance itself was close to ceiling for humans.

**Figure 3:**
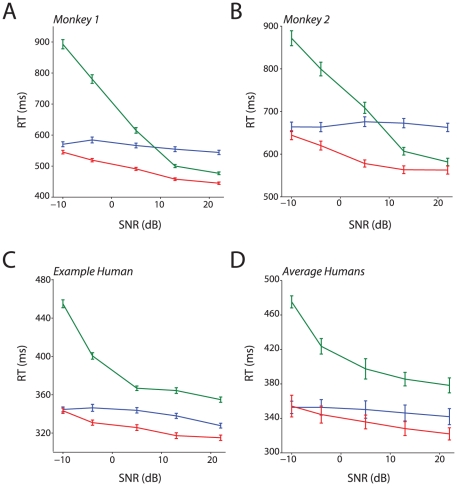
RTs to Auditory, visual and audiovisual vocalizations. **A**: Mean RTs obtained by pooling across all sessions as a function of SNR for the unisensory and multisensory conditions for Monkey 1. Error bars denote standard error of the mean estimated using bootstrapping. X-axes denote SNR in dB. Y-axes depict RT in milliseconds. **B**: Mean RTs obtained by pooling across all sessions all sessions as a function of SNR for the unisensory and multisensory conditions for Monkey 2.Conventions as in A. **C**: Mean RTs obtained by pooling across all sessions as a function of SNR for the unisensory and multisensory conditions for a single human subject. Conventions as in A. **D**: Average RT across all human subjects as a function of SNR for the unisensory and multisensory conditions. Error bars denote SEM across subjects. Conventions as in A.

In both species, the similarities in detection accuracy for visual-only and audiovisual conditions (Figures 2A–D) suggest that they were perhaps not integrating auditory and visual signals but instead may have adopted a unisensory (visual) strategy. According to this strategy, they used visible mouth motion only for both the visual and audiovisual conditions, and used the sound only when forced to do so in the auditory-only condition. We therefore examined the reaction times (RTs) to distinguish between a unisensory versus an integration strategy. Figures 3A, B show the mean RT as a function of the SNR and modality computed by pooling RT data from all the sessions for Monkeys 1 and 2. RTs for the auditory-only vocalization increased as the SNR decreased (i.e. the sound was harder to hear relative to the background). In contrast, RTs to the visual-only condition only increased weakly with an increase in the mouth opening size — a result consistent with the accuracy data. Although the audiovisual accuracy was only modestly better than the visual-only accuracy (Figure 2A,B), audiovisual RTs decreased relative to both auditory-only and visual-only RTs for several SNR levels. To illustrate, a non-parametric ANOVA (Kruskal-Wallis) computed for Monkey 1, which compared the ranks of the RTs for the auditory-only, visual-only and audiovisual conditions for the highest SNR (+22dB), was significant (χ^2^ = 490.91, p<.001). Post-hoc Mann-Whitney U tests revealed that the RT distribution for the audiovisual condition was significantly different from the auditory-only distribution and the visual-only distribution for all SNRs; that is, RTs in the audiovisual condition were faster than visual and auditory RTs. In Monkey 2, the audiovisual RT distribution was different from the auditory-only distribution for all SNRs (p<0.001), and was significantly different from the visual-only distribution for all but the lowest SNR (−10 dB, p = 0.68). It is notable that at the highest SNR (+22 dB; largest mouth opening), the RTs of both monkeys seem more like the auditory-only RTs, while at the lowest SNR (−10 dB; smallest mouth opening), the RTs seem to be more similar to the visual-only RTs.

Humans also show a RT benefit in the audiovisual versus unisensory conditions, with a similar, but not identical, pattern to that observed in monkeys. Figure 3C shows the average RTs of a single human subject as a function of the SNR. Similar to monkeys, decreasing the SNR of the auditory-only condition leads to an increase in the RTs, and RTs for the visual-only condition were only weakly modulated by the size of the mouth opening. For a range of SNRs, the audiovisual RTs were faster than auditory- and visual-only RTs. Figure 3D shows the average RTs over all 6 subjects. Paired t-tests comparing audiovisual RTs to auditory-only and visual-only RTs reveal that they were significantly different in all but the lowest SNR condition (p = 0.81 for the −10 dB condition, p<0.05 for all other conditions, df  = 5). Though the RT patterns from human participants seem dissimilar to the monkey RT patterns (e.g., in monkeys the auditory-RT curve crossed the visual-only RT curve but for humans there was no cross over), we can show that the two species are adopting a similar strategy by exploring putative mechanisms. We do so in the next sections.

### A race model cannot explains benefits for audiovisual vocalizations

Our analysis of RTs rules out the simple hypothesis that monkeys and humans are defaulting to a unisensory strategy (using visual in all conditions except when forced to use auditory information). Another hypothesis is that a “race” mechanism is at play [59]. A race mechanism postulates parallel channels for visual and auditory signals that compete with one another to terminate in a motor or decision structure and thereby trigger the behavioral response. We chose to test this model to ensure that the observers were actually integrating the faces and vocalizations of the avatar. A simple physiological correlate of such a model would be the existence of independent processing pathways for the visual mouth motion and an independent processing pathway for the auditory vocalization. In the race scenario, there would be no cross-talk between these signals. Race models are extremely powerful and are often used to show independent processing in discrimination tasks [70], [71], [72]. In our task, independent processing would mean that in the decision structure, two populations of neurons received either auditory or visual input. These two independent populations count spikes until a threshold is reached; the population that reaches threshold first triggers a response. Such a model can lead to a decrease in the RTs for the multisensory condition, not through integration, but through a statistical mechanism: the mean of the minimum of two distributions is always less than or equal to the minimum of the mean of two distributions.

**Figure 4:**
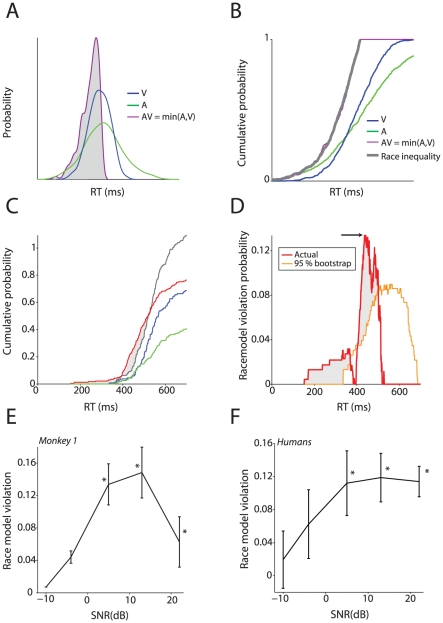
Race models cannot explain audiovisual RTs. **A:** Schematic of a race mechanism for audiovisual integration. The minimum of two reaction time distributions is always faster and narrower than the individual distributions. **B**: Race models can be tested using the race model inequality for cumulative distributions. The graph shows the cumulative distributions for the density functions shown in A along with the race model inequality. **C**: Cumulative distributions of the auditory, visual and audiovisual RTs from monkey 1 for one SNR (+5dB) and one inter stimulus interval (ISI) window (1000 – 1400 ms) along with the prediction provided by the race model. X-axes depict RT in milliseconds. Y-axes depict the cumulative probability. **D**: Violation of race model predictions for real and simulated experiments as a function of RT for the same SNR and ISI shown in C. X-axes depict RT in milliseconds. Y-axes depict difference in probability units. **E**: Average race model violation as a function of SNR for the ISI of 1000 to 1400 ms for Monkey 1. Error bars denote the standard error estimated by bootstrapping. * denotes significant race model violation using the bootstrap test shown in D. **F**: Average race model violation across human subjects as a function of SNR. X-axes depict SNR; y-axes depict the amount of violation of the race model in probability units. * denotes significant race model violation according to the permutation test.

Figure 4A shows a simulation of this race model. The audiovisual distribution, if it is due to a race mechanism, is obtained by taking the minimum of the two distributions and will have a lower mean and variance compared to the individual auditory and visual distributions. Typically, to test if a race model can explain the data, cumulative distributions of the RTs (Figure 4B) are used to reject the so-called race model inequality [51], [57]. The inequality is a strong, conservative test and provides an upper bound for the benefits provided by any class of race models. Reaction times faster than this upper bound mean that the race model cannot explain the pattern of RTs for the audiovisual condition; the RT data would therefore necessitate an explanation based on integration.

Figure 4C plots the cumulative distributions for RTs collected in the intermediate SNR level and for ISIs between 1000 and 1400 ms for Monkey 1; the prediction from the race model is shown in grey. We used this ISI interval because, in monkeys only, the ISI influenced the pattern of audiovisual benefits (**see Text S1 and Figure S2**). Maximal audiovisual benefits were for ISIs in the 1000–1400 ms range. The cumulative distribution of audiovisual RTs is faster than can be predicted by the race model for multiple regions of RT distribution, suggesting that the RTs cannot be fully explained by this model. To test whether this violation was statistically significant, we compared the violation from the true data to one using conservative bootstrap estimates. Several points for the true violation were much larger than the violation values estimated by bootstrapping (Figure 4D). Audiovisual RTs are therefore not explained by a race model. For the entire range of SNRs and this ISI for the monkeys, maximal race model violations were seen for the intermediate to high SNRs (+5, +13 and + 22 dB; Figure 4E). For the softer SNRs (−10,−4 dB), a race model could not be rejected as an explanation. The amount of race model violation for the entire range of ISIs and SNRs is provided in **Figure S3**. For both monkeys, longer ISIs resulted in weaker violations of the race model and rarely did the p-values from the bootstrap test reach significance.

For humans, we observed similar robust violations of the race model. Figure 4F shows the average amount of race model violation across subjects as a function of SNR. Since humans showed much less dependence on the ISI, we did not bin the data as we did for monkeys. Similar, to monkeys, maximal violation of the race model was seen for loud and intermediate SNRs. For 3 out of the 5 SNRs (+22, +13, +5 dB), a permutation test comparing maximal race model violation to a null distribution was significant (p<0.05). In conclusion, for both monkeys and humans, a race model cannot explain the pattern of RTs at least for the loud and intermediate SNRs.

These results strongly suggest that monkeys do *integrate* visual and auditory components of vocalizations and that they are similar to humans in their computational strategy. In the next sections, we therefore leveraged these behavioral data and attempt to identify a homologous mechanism(s) that could explain this pattern of results. Our search was based on the assumption that classical principles and mechanisms of multisensory integration [48], [49], [50], [51], [73], originally developed for simpler stimuli, could potentially serve as starting hypotheses for a mechanism mediating the behavioral integration of the complex visual and auditory components of vocalizations.

### Mechanism/Principle 1: Principle of inverse effectiveness

**Figure 5:**
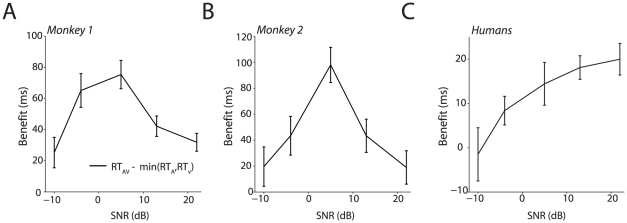
Benefit in RT for the audiovisual condition compared to unisensory conditions. **A**: Mean benefit in RT for the audiovisual condition relative to the minimum of mean visual-only and auditory-only RTs for monkey 1. X-axes depict SNR. Y-axes depict the benefit in milliseconds. Error bars denote standard errors estimated through bootstrap. **B**: Mean benefit in RT for the audiovisual condition relative to the minimum of mean visual-only and auditory-only RTs for monkey 2. Conventions as in A. **C:** Mean benefit in RT for the audiovisual condition relative to the minimum of the mean visual-only and auditory-only conditions averaged across subjects. Axis onventions as in A. Error bars denote standard errors of the mean.

The first mechanism we tested was whether the integration of faces and voices demonstrated in our data followed the “principle of inverse effectiveness” [49], [50]. This idea, originally developed to explain neurophysiological data, suggests that maximal benefits from multisensory integration should occur when the stimuli are themselves maximally impoverished [49], [50], [74], [75]. That is, the weaker the magnitude of the unisensory response, the greater would be the gain in the response due to integration. In our case with behavior, this principle makes the following prediction. As the RTs and accuracy were the poorest for the lowest auditory SNR, the benefit of multisensory integration should be maximal when the lowest auditory SNR is combined with the corresponding mouth opening. Our metric for multisensory benefit was defined as the speedup for the audiovisual RT relative to the fastest mean RT in response to the unisensory signal (regardless of whether it was the auditory- or visual-only condition). The principle of inverse effectiveness would thus predict greater reaction time benefits with decreasing SNR for both monkeys and humans. Figures 5A and B plot this benefit as a function of SNR for Monkeys 1 and 2. For monkeys, the maximal audiovisual benefit occurs for intermediate SNRs. The corresponding pattern of benefits for humans is shown in Figure 5C. For humans, this benefit increases as the SNR increases and starts to flatten for the largest SNRs. This pattern of benefits reveals that the maximal audiovisual RT benefits do not occur at the lowest SNRs. This is at odds with the principle of inverse effectiveness [49], [50]. If our results had followed this principle, then the maximal benefit relative to both unisensory conditions should have occurred at the lowest SNR (lowest sound intensity coupled with smallest mouth opening). Neither monkey nor human RTs followed this principle and therefore it cannot be a homologous mechanism mediating the integration of faces and voices in primates.

One potential caveat is that we are testing the principle of inverse effectiveness using absolute reaction time benefits whereas the original idea was developed using proportional referents. Thus, we re-expressed the benefits as a percent gain relative to the minimum of the auditory and visual reaction times for each SNR. We observed that, even when converted to a percent benefit relative to the minimum reaction time for each SNR, the inverted U-shape pattern of gains for monkeys (**Figures S4A, B**), as well as increasing gain with SNR for humans (**Figure S4C**), was replicated. Thus, whether one uses raw benefits or a proportional measure, RT benefits from combining visual and auditory signals could not be explained by invoking the principle of inverse effectiveness.

### Mechanism/Principle 2: Physiological synchrony

If inverse effectiveness could not explain our results, then what other mechanism(s) could explain the patterns of reaction time benefits? Monkey performance at intermediate SNRs (where the maximal benefits were observed; Figures 3A, B), the visual-only and auditory-only reaction time values were similar to each other. Similarly, for humans at intermediate to large SNRs (where maximal benefits were observed for humans), the visual-only and auditory-only reaction time values were similar to one another. This suggests a simple timing principle: the closer the visual-only and auditory-only RTs are to one another, the greater is the multisensory benefit. A similar behavioral result has been previously observed in the literature, albeit with simpler stimuli, and a mechanism explaining this behavior was (somewhat confusingly) dubbed “physiological synchrony” [51], [73]. According to this mechanism, developed in a psychophysical framework, performance benefits for the multisensory condition are modulated by the degree of overlap between the theoretical neural activity patterns (response magnitude and latency) elicited by the two unisensory stimuli [51], [73]. Maximal benefits occur during “synchrony” of these activity patterns; that is, when the latencies overlap. To put it another way, maximal RT benefits will occur when the visual and auditory inputs arrive almost at the same time.

**Figure 6:**
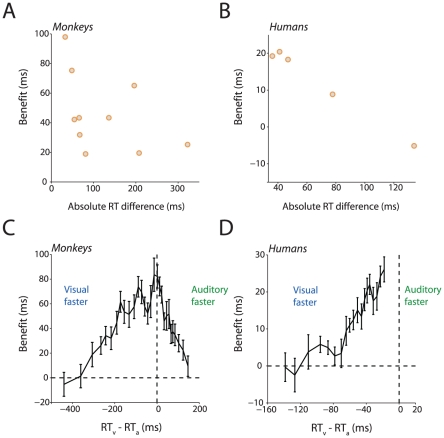
Time window of integration. **A:** Reaction time benefits for the audiovisual condition in monkeys decrease as the absolute difference between visual-only and auditory-only RTs decrease. X-axes depict difference in ms. Y-axes the benefit in milliseconds. **B:** Reaction time benefits for the audiovisual condition in humans also decrease as the absolute difference between visual-only and auditory-only RTs decrease. Conventions as in A. **C**: Mean benefit in the RT for the audiovisual condition relative to minimum of the auditory-only and visual-only RTs as a function of the difference between mean visual-only and auditory-only RTs for monkey 1. X-axes depict reaction time difference in ms. Y-axes depict benefit in ms. **D**: Mean benefit in the RT for the audiovisual condition relative to minimum of the auditory-only and visual-only RTs as a function of the difference between mean visual-only and auditory-only RTs for humans. Conventions as in C.

To test this idea, we transformed the benefit curves shown in Figures 5A-C by plotting the benefits as a function of the absolute value of the difference between visual-only and auditory-only RTs. That is, instead of plotting the benefits as a function of SNR (as in Figures 5A–C), we plotted them as a function of the difference between the visual-only and auditory-only RTs for each SNR. If our intuition is correct, then the closer the auditory- and visual-only RTs are (i.e., the smaller the difference between them), then the greater would be the benefit. Figure 6A plots the benefit in reaction time as a function of the absolute difference between visual- and auditory-only RT for monkeys 1 & 2. The corresponding plot for humans is shown in Figure 6B. By and large, as the difference between RTs increase, the benefit for the audiovisual condition decreases with the minimum benefit occurring when visual- and auditory-only RTs differ by more than 100 to 200 milliseconds. Thus, physiological synchrony can serve as a homologous mechanism for the integration of faces and voices in both monkeys and humans.

Although the original formulation of the principle suggested “synchrony”, it seemed too restrictive. The reaction time data—at least for integrating faces and voices—suggest that there is a range of reaction time differences over which multisensory benefits can be achieved. That is, there is a “window of integration” within which multisensory benefits emerge. We use the term “window of integration” as typically defined in studies of multisensory integration: It is the time span within which auditory and visual response latencies must fall so that their combination leads to behavioral or physiological changes significantly different from responses to unimodal stimuli. Such windows have been demonstrated in physiological [49], [76] as well as in psychophysical studies of multisensory integration[48], [77]. To explore the extent of this “window of integration”, we elaborated upon the analysis shown in Figures 6A and B to the whole dataset of sessions and SNRs. For all the sessions and SNRs (48 sessions and 5 SNRs for 2 monkeys), we computed a metric that was the difference between the mean visual-only and auditory-only RTs. This gave us 480 values where there was a difference between visual and auditory RTs and, corresponding to this value, the benefit for the audiovisual condition. After sorting and binning these values, we then plotted the audiovisual benefit as a function of the difference between the mean visual-only and auditory-only RTs. Figure 6C shows this analysis for monkeys. Only in an intermediate range, where differences between unisensory RTs are around 100 – 200 ms, is the audiovisual benefit non-zero—with a maximal benefit occurring at approximately 0 ms. In addition, this window is not symmetrical around zero. It is 200 ms long when visual RTs are faster than auditory RTs and around 100 ms long when auditory-only RTs are faster than visual-only RTs. We repeated the same analysis for humans and the results are plotted in Figure 6D. For humans, a similar window exists: when visual reaction times are faster than auditory reaction times then the window is approximately 160 ms long. We could not determine the extent of the window because, in humans, auditory RTs were never faster than visual RTs.

To summarize, combining visual and auditory cues leads to a speedup in the detection of audiovisual vocalizations relative to the auditory-only and visual-only vocalizations. Our analysis of the patterns of benefit for the audiovisual condition reveals that maximal benefits do not follow a principle of inverse effectiveness. However, the principle of physiological synchrony that incorporates a time window of integration provided a better explanation of these results.

### Mechanism/Principle 3: A linear superposition model

The principle of physiological synchrony with a time window of integration provides an insight into the processes that lead to the integration of auditory and visual components of communication signals. The issue however is that although this insight can be used to predict behavior, it does not have any immediate mechanistic basis. We therefore sought a computational model that could plausible represent the neural basis for these behavioral patterns. We specified two criteria for the model based on our results. First, audiovisual RTs should be faster than auditory- and visual-only RTs. Second, it should be consistent with, and perhaps subsume, the principle of physiological synchrony with a time window of integration—benefits accrued by combining visual and auditory cues should occur when the visual- and auditory-only RTs are almost equal to one another. If these two criteria are validated, then the model would be a straightforward homologous mechanism.

**Figure 7:**
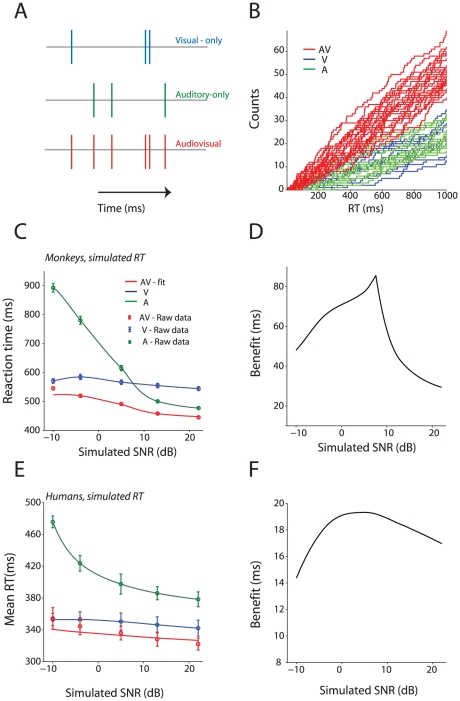
Superposition models can explain audiovisual RTs. **A:** Illustration of the superposition model of audiovisual integration. Ticks denote events which are registered by the individual counters. **B:** Simulated individual trials from the audiovisual, auditory-only and visual-only counters. X-axes denotes RT in milliseconds, y-axes the number of counts. **C:** Simulated and raw mean RTs using parameters estimated from the visual-only and auditory-only conditions for monkey 1. X-axes denote simulated SNR in dB. Y-axes denote RTs in ms estimated using a superposition model. The raw data are shown as circles along with error bars. The estimated data for the audiovisual condition is shown in a red line. **D:** Simulated benefits for audiovisual RTs relative to the auditory-only and visual only conditions as a function of SNR. Note how the peak appears at intermediate SNRs. **E:** Simulated and raw mean RTs using parameters estimated from the real visual- and auditory-only conditions for humans. X-axes denote simulated SNR in dB. Y-axes denote RTs in ms estimated using a superposition model. The raw data are shown as circles along with errorbars. The estimated data for the audiovisual condition is shown in red. Conventions as in C. **F:** Simulated benefits for human audiovisual RTs relative to the auditory-only and visual only conditions as a function of SNR, note how as in real data, benefit increases with increasing SNR and plateaus for large SNRs. Conventions as in D.

Superposition models are one class of integration models that could incorporate our criteria [53], [63], [64]. According to these models, activation from different sensory channels is linearly combined until it reaches a criterion/threshold and thereby triggers a response. We will use a model formulation based on counters for simplicity [63]. According to this counter model, the onset of a stimulus would lead to a sequence of events occurring randomly over time. Let N (*t*) denote the number of events that have occurred by time *t* after stimulus presentation. After the number of counts reaches a criterion, *c*, it triggers a response. Let us assume that there are separate counters for visual and auditory conditions, N_V_ (*t*) and N_A_ (*t*). During the audiovisual condition, a composite counter, N_AV_ (*t*)  =  N_A_ (*t*) + N_V_ (*t*), comprised of both the visual and auditory signals, counts to the criterion, *c* (Figure 7A). This composite, multisensory counter would reach the criterion faster than either of the unisensory counters alone. Figure 7B shows that a computer simulation of a counter composed of superposed activity from both visual and auditory cues would reach criterion faster than the unisensory ones alone.

Using the RT data from Monkey 1, we set the parameters of the superposition model for the auditory- and visual-only RTs and then used the model to estimate the audiovisual RTs (Figure 7C). From this, the model produced audiovisual RTs that were faster than both the auditory-only and visual-only RTs—like the pattern of results we observed for monkeys (Figures 3A, B). As Figure 7C shows, except for the lowest SNR, there is a good one to one correspondence between the model's prediction of audiovisual RTs and the actual raw data. Thus, this model can at least generate the patterns of reaction times observed in response to audiovisual vocalization.

We next estimated the benefits in RT for the audiovisual condition relative to the visual-only and auditory-only condition from the simulated model (Figure 7D). The benefit curve has the same inverted U-shaped profile as the real patterns of benefit shown in Figure 5A. We repeated this analysis for the human RTs and the pattern of results is shown in Figure 7E–F. Figure 7E shows the predicted reaction time of the average participant as a function of SNR along with actual data. The predicted reaction times are very similar to the actual RTs observed for humans in Figure 3D. As with the monkey behavioral data, the fits performed worst for the softest SNR. Like the benefit patterns shown in Figure 5C, the benefit for the AV condition increases as SNR increases (Figure 7F). This replication by the model of the pattern of monkey and human data—faster audiovisual RTs and maximal benefit when visual and auditory RTs are well matched—suggests that a superposition model is a viable homologous mechanism.

## Discussion

The goal of our study was threefold. First, do monkeys integrate the visual and auditory components of vocalizations? Second, is monkey behavior similar to that of humans perfoming an identical task? Third, is there a homologous mechanism for the processing of audiovisual communication signals? We trained monkeys and asked humans to detect vocalizations by monkey and human avatars, respectively, in a noisy background. We measured their accuracy and reaction times. We found that monkeys do integrate the visual and auditory components of vocalizations (as measured by faster reaction times for the audiovisual relative to the unisensory conditions). Similar speedups in reaction times were observed also for human subjects. Rejection of the race model demonstrated that the behavioral patterns must be explained by an integrative process (one requiring the use of both unisensory channels together to drive behavioral change), and not one based on competing independent unisensory channels. We then tested whether classical principles of multisensory integration could serve as homologous mechanisms for the integration of faces and voices. The “principle of inverse effectiveness” failed to explain the data for either primate species. Both monkey and human RTs were better explained by the principle of “physiological synchrony” that incorporated a time window of integration. We found that a simple computational model positing the linear superposition of activity induced by visual and auditory cues could explain the pattern of results in monkeys as well as humans. Critically, its explanatory power was such that it could explain the small differences in behavior observed for monkeys and humans. Furthermore, the superposition model is completely consistent with the principles of physiological synchrony with a time window of integration. The superposition model, therefore, is an excellent candidate for a homologous mechanism used by monkeys and humans to integrate faces and voices.

### Monkeys like humans can integrate visual and auditory components of vocalizations

Monkeys and humans share many homologous mechanisms for the production of vocalizations [22]. In humans, these production mechanisms deform the face in such a manner that facial motion enhances the detection and discrimination of vocal sounds by receivers [6], [7], . Often this enhanced behavior takes the form of decreased reaction times to audiovisual versus unisensory presentations of speech [8], [9]. While nonhuman primates could theoretically use the same or very similar facial motion to enhance their auditory perception, there has been no evidence of this to date. Several studies demonstrated that, like human infants, monkeys and apes can match facial expressions to vocal expressions [26], [27], [28], [29], and that eye movement patterns generated by viewing vocalizing conspecifics is similar between monkeys and humans [30], [31], [32]. None of these nonhuman primate studies, however, demonstrated a *behavioral advantage* for perceiving audiovisual vocalizations over unisensory expressions. Demonstration of such an advantage is necessary to invoke the hypothesis that a multisensory integration mechanism for communication signals is homologous across species. In the current study, we provide the first demonstration that monkeys exhibit a behavioral advantage for audiovisual versus unisensory presentations of vocalizations. The patterns of both accuracy and reaction time benefits were similar to humans performing an identical task.

Although we have emphasized throughout the similarities in the patterns of behavior for monkeys and humans, it is important to note that there were also differences. The most important difference was that humans were consistently faster for the visual-only vocalization compared to the auditory-only vocalization across the range of auditory intensities. Monkeys, on the other hand, responded faster to some auditory-only conditions versus visual-only conditions across the range of intensities. These differences ultimately led to differences in the amount of integration. Such differences could potentially arise due to the differences in auditory stimuli (/u/ sounds in humans vs coo calls in monkeys) or the amount of attentional engagement. We have suggested acoustic equivalence of “coos” and /u/ vocalizations, but they are not communicatively equivalent. Coos are common vocalizations in monkeys with behavioral significance including a positive emotional valence. In contrast, the /u/ sound we used with humans does not have any behavioral significance. With regard to engagement, we trained our monkeys using standard operant conditioning techniques. This meant the use of timeouts as negative reinforcement whenever the monkeys made false alarms. As a result, when compared to human performance, monkeys may adopt a more conservative criterion for the detection of these sounds to avoid false alarms. Despite these caveats, it is worth emphasizing that positing a linear superposition of visual and auditory signals reconciled these dissimilar results from the two species.

Two other design features of our study are worth pointing out before we discuss the broader implications of our results. First, we used a fixed delay between the mouth motion and the onset of the vocalization. Under natural conditions, delays between onset of mouth opening and sound onset, which we term time-to-voice (TTV), are wide ranging and can vary from utterance to utterance and speaker to speaker [5]. At the neural level, different TTVs modulate the degree of integration in local field potential signals recorded from the upper bank of the superior temporal sulcus of monkeys [40]. Thus, how this variable would affect behavioral integration of faces and voices in monkeys and humans is not tested in our experiments or in any other study.

A second design feature that we used consisted of the presence of a static face on the screen during the auditory-only vocalization. This face was also identity-incongruent with the auditory vocalization. Thus, both of these features could potentially *slow down* auditory-only RTs by creating confusion: the face doesn't move when it should during a vocalization and/or the face doesn't match the identity of the voice. However, we believe this concern is mitigated by the more naturalistic conditions that our design mimics and more pressing problems that it avoids. Our paradigm is naturalistic in the following sense: faces in noisy, cocktail-party like scenarios do not appear and disappear. Furthermore, monkeys like humans can recognize indexical cues in vocalizations (cues that indicate body size, age, identity, etc) and match them to faces [82], [83]. Thus, in our paradigm, it is not odd to hear one individual's voice while seeing another individual's face, a typical occurrence under natural conditions. The key to the face-voice integration is combining motion of the face to the correct, corresponding voice. If we did not present a static face during the auditory-only condition and observed an audiovisual benefit, then the benefits could be attributed to differences in overall attention or arousal (a frequent criticism of physiological studies of AV integration). Moreover, if we adopted a design where audiovisual vocalizations involved the sudden onset of a face followed by its mouth motion, then any RT benefits for audiovisual compared to auditory-only vocalizations would be uninterpretable: we could not be sure if it was due to the integration of facial motion with the sound or from the integration of the sound with the sudden onset of the face.

Whatever influences our design may actually have on our participants' RTs; we can model the outcome of hypothetically faster RTs that may arise with a study design that did not use a static, incongruent face in the auditory-only conditions. Since our data demonstrate that the principle of physiological synchrony with a time window of integration, we can actually perform a thought experiment to see what would happen if our auditory RTs are sped up. Simply put, the result would be that the point at which visual and auditory RT curves cross will be at a different SNR and this point of crossing would be the new point of maximal integration. **Figure S5** shows that if we sped up all auditory RTs by 40, 80 and 120 ms in the model relative to the original data, the point of maximal integration shifts to lower SNRs.

### Integration of complex versus simple multisensory signals

We demonstrated that combining visual mouth motion with auditory vocalizations speeds up reaction times in monkeys and humans. Faster reaction times to multisensory signals compared to unisensory signals are a frequent outcome in human psychophysical studies [51], [57], [58], [59], [84], [85], [86], [87]. The first such demonstration, nearly a hundred years ago, showed that there was a speedup in responses for bi- and tri-modal stimuli compared to unimodal stimuli [87]. Since then, this seminal result has been replicated in a variety of settings almost always with the use of simple stimuli [51], [57], [85], [88], [89], [90], [91]. In particular, shortened reaction times are observed in response to multimodal stimuli using both saccades and lever presses as dependent measures [85], [92]. Physiologically, there are similar results. Neurons in the superior colliculus of anaesthetized cats respond faster to audiovisual compared to auditory and visual stimuli [93]. Our results confirm that similar behavioral advantages exist when combining the visual and auditory components of complex social signals encountered in everyday settings.

While there are certainly similarities in the integration processes for simple and complex signals like speech, there are also differences. An important issue which has been repeatedly demonstrated is that there are differences in the window of integration for simple versus complex stimuli[94]. For the integration of simple stimuli, tolerance of asynchrony between visual and auditory cues is very small leading to a narrow window of integration [94]. In contrast, for speech stimuli, observers are able to tolerate very large asynchronies and still bind them into a common percept[47]. We return to this issue later in the Discussion.

### Behavioral detection of audiovisual communication signals cannot be explained by the principle of inverse effectiveness

For both monkeys and humans, we found that the maximal benefit obtained by combining visual and auditory cues was for *intermediate* values of SNR. This is at odds with the principle of inverse effectiveness [49], [50]. This idea was originally formulated in the context of electrophysiological experiments and suggests that the maximal benefit (greater proportional response magnitude) from multisensory stimulus inputs would be achieved by combining visual and auditory cues that, individually, elicit weak responses. Support for the inverse effectiveness rule is also evident at the behavioral level in both monkeys and humans in detection tasks involving simple stimuli [85], [92], [95], [96]. If this principle held true for detecting vocalizations, then we would have observed maximal reaction time savings for the lowest SNR, with the benefit decreasing with increasing SNR. On the contrary, monkeys' detection of vocalizations generated a non-monotonic curve with peak multisensory benefits occurring at intermediate SNRs. For humans, the multisensory benefit increased with increasing SNRs. Thus, for the multisensory integration of vocalizations (with reaction times as a behavioral measure), neither in monkeys nor in humans does the principle of inverse effectiveness explain the behavior. Other results from the speech processing literature support our assertion. For example, in studies of speech intelligibility, maximal benefits gained by integration of auditory speech with visual speech are found when the auditory speech is presented in an intermediate, versus high, level of noise [7], [81]. Similarly, the McGurk effect occurs even under clear listening conditions (i.e., noisy signals aren't required to generate the illusory percept) [10], and vision can boost the comprehension of extended auditory passages even under excellent listening conditions [97].

As mentioned before, there are several studies which claim to support this principle in behavior [75], [85], [92], [95], [96], [98], [99], [100], so why do we not see support for the principle of inverse effectiveness in our data or in other studies [51], [57], [88], [92], [101]? We think that this principle is sensitive to the way multisensory stimuli are parameterized and tested in different experiments. In particular, the choice of stimuli, levels of intensity and the pairing of stimuli could all affect whether this principle will be apparent in the resultant data. To illustrate what we mean, we tested two hypothetical scenarios, where inverse effectiveness can be observed using RTs and compare it to a scenario resembling our experimental data. For each scenario, we constructed auditory and visual RTs to have a certain profile with respect to different intensity levels. Then, given that the superposition model is an excellent explanation of our RT data, as well as RTs to simple stimuli[53], [62], [63], [64], [86], we used it to simulate the expected audiovisual reaction times for these same intensity levels. We then examined if the multisensory benefits were consistent with the principle of inverse effectiveness or not. The first scenario is a case wherein RTs to both senses increase with decreases in intensity level, but at every intensity level, they are still roughly equal to one another (**Figure S6A**). In this scenario, RTs to visual and auditory stimuli increase with decreasing intensity and visual and auditory RTs are largely similar at every intensity level. Keeping with multisensory integration, audiovisual RTs are faster than both auditory-only and visual-only RTs. Critically, in line with our intuition, the multisensory benefit increases with the decrease in SNR — and is thus consistent with the principle of inverse effectiveness (**Figure S6B**).

We can also outline a second scenario where this principle would be observed to be in action. This is the case when the stimuli are such that the RT of one modality approaches the RT of the other modality only for the lowest intensity levels. **Figure S6C** shows a simulation of this scenario. The auditory-only RTs are much faster than the visual-only RTs for the highest intensity levels. However, as the stimulus intensity decreases, the auditory- and visual-only RTs approach each other. Again, audiovisual RTs are faster than auditory- and visual–only RTs. Like the previous scenario, as intensity decreases, the benefit increases and is thus consistent with the principle of inverse effectiveness (**Figure S6D**). A recent study showing support for inverse effectiveness had visual and auditory RTs closely following this scenario [99]. The third scenario is one that is a simulation of our data (**Figure S6E**). In this case, visual RTs do not change much with intensity level, but auditory RTs increase with a decrease in intensity. Audiovisual RTs are again faster than auditory and visual-only RTs. Critically, these data result in a pattern of benefits that is non-monotonic and takes the form of an inverted U; it is not consistent with the principle of inverse effectiveness (**Figure S6F**).

In summary, given that the superposition model is an excellent fit to data, simulations of this model using the scenarios above suggest that observing the principle of inverse effectiveness in behavior is to some extent dependent upon the way the parameters of the stimuli that are used in an experiment. Different multisensory stimuli (speech versus non-speech) as well as the choice of intensity levels are bound to have different effects on multisensory benefit. Thus, the principle of inverse effectiveness may be operational only under some situations. We would however note that, this framework of superposition only explains the inconsistencies about inverse effectiveness in RT output. A similar careful analysis is needed to explain accuracy of subjects as well as performance in tasks such as localization [75], [98].

### Audiovisual integration of communication signals adheres to the principle of “physiological synchrony” with a time window of integration

We showed that maximal benefits from integration of visual and auditory components of communication signals occurred when the reaction times to visual and auditory cues are themselves very similar to one another. This is consistent with the idea of “physiological synchrony”, a principle proposed to explain behavioral data. The principle of physiological synchrony was first formulated based on psychophysical experiments using punctate, simple stimuli [51], [73]. In these experiments, it was noted that maximal multisensory benefits occurred when the stimulus-onset asynchrony between visual and auditory stimuli was adjusted to be equal to the difference between visual-only and auditory-only RTs. That is, “synchrony” was defined by theoretical neurophysiological activity (with reaction times as a proxy) rather than physical synchrony defined by the stimulus-onset asynchrony. According to this idea, performance benefits for the multisensory condition are modulated by the degree of temporal overlap between the theoretical neurophysiological activity patterns elicited by the two unisensory stimuli [51], [73]. Maximal benefits occur during synchrony of these neural activity patterns; that is, when their latencies over-lap.

It is worth repeating that this notion of physiological synchrony is a behavioral construct derived by considering RTs. RTs are a simple but powerful metric for indexing this behavior. However, they are the output of a complex mixture of sensory processing, motor preparation, temporal expectation, attention and other cognitive processes. Thus, the physiological synchrony mechanism, although it explains patterns of behavior using RTs to sensory stimuli does not necessarily predict that the integration is occurring in a purely sensory circuit. The neural locus where integration is taking place is not known. Sensory, premotor and/or motor circuits involved in multisensory processing are very likely all involved in generating behavioral responses during this task.

We found that there was a time window within which differences in reaction times between visual and auditory signals could lead to integration. This notion of a “temporal window of integration” is a recurring concept in behavioral and neurophysiological experiments of multisensory integration [48], [76], [77], [84], [89], [102], [103]. For example, participants perceive the McGurk effect when the stimulus-onset asynchrony between visual and auditory cues is in a window approximately 400 milliseconds wide, beyond which the illusion disappears [48]. Similarly, studies of orienting responses to audiovisual stimuli using saccades show that speedup of saccadic RTs occur in a variety of experimental settings within a time window of 150–250 ms [77], [84], [89], [104], [105]. Finally, neurophysiologically, maximal integration in multisensory neural responses in the superior colliculus is observed when the stimulus onset asynchrony is adjusted such that the discharge patterns to visual and auditory signals themselves overlap with each other [76].

### A linear superposition model of integration is a putative homologous mechanism

We showed that a simple computational model of integration—a linear superposition model—explained the behavioral patterns observed for the integration of audiovisual vocalizations by monkeys and humans. The main tenet of this model is that the information from the two unisensory channels is integrated at a specific processing stage by the linear summation of channel-specific activity patterns. Superposition models have been successfully used to predict the reaction times of observers in other multisensory detection tasks, albeit with much simpler stimuli [53], [62], [63], [64], [86]. Physiologically, support for this principle was suggested in studies of the sensitivity of multisensory neurons in superior colliculus [76]. Our results suggest that this model can be readily extended to the integration of visual and auditory components of vocalizations, at least during behaviors involving speeded detection. Indeed, invoking this mechanism reconciled the observed dissimilarity in RTs from monkeys and humans. In addition, it automatically subsumes the principle of physiological synchrony and generates appropriately asymmetric time windows of integration. Whether this model works well for other tasks such as multisensory spatial orientation [75], [98], is an open question. Nevertheless, for the task presented in this study, i.e. the detection of vocalizations in noise, it is a parsimonious homologous mechanism.

That a linear, additive model could provide a good explanation for the detection of audiovisual vocalizations might seem irreconcilable with typical notions of multisensory integration that emphasize “super-additivity” or non-linear responses [49], [50]. Recent studies, however, report that multisensory neurons can integrate their inputs in an additive manner both in terms of spiking activity [See for e.g. 50,52,106], as well at the level of synaptic input [107]. Our emphasis on the superposition model as a homologous mechanism has another important implication. First, there are a remarkable number of nodes on which visual and auditory inputs that are sensitive to faces and voices, respectively, could converge. Any or all of these sites could be responsible for the behavioral advantage we report here. For example, neurons in the amygdala and association areas such as the upper bank of STS and prefrontal cortex respond to both the visual and auditory components of vocalizations. In some cases, we know that they integrate these vocalization-related cues [40], [42], [43], [108], [109]—at least during the passive reception of these signals. For example, in keeping with the linear superposition model we posited here, approximately 7% of ventrolateral prefrontal cortical neurons integrate visual and auditory components of vocalizations linearly [43].

The superposition model subsumes the time window of integration. The basis of superposition models is that they require activity patterns to overlap with one another and add together to generate benefits. Thus, activity patterns that overlap with one another have a higher probability of leading to integration, whereas activity patterns that do not overlap will not lead to integration. This implies that the measured window of integration is going to depend on the inherent statistics of the visual and auditory signals and the response profiles to the two signals in some neural structure on which they converge. The narrowness and the latency of these response profiles will thus determine the window of integration. Thus, in any given experiment, choices of the strength and duration of these visual and auditory signals would automatically result in corresponding changes in latencies and response profiles. A flash is highly likely to be processed in primary visual cortex and a moving face through a combination of face- and motion-sensitive neural structures. A similar argument can be made for auditory stimuli. Thus, unless the response profile(s) in some integrative structure(s) mediating detection of these various stimuli are identical, the windows of integration are bound to be different for simple stimuli such as flashes and tone pips versus more complex audiovisual vocalizations and speech signals. This might be a partial explanation for one of the best known findings in the multisensory literature — asymmetric broad windows for speech [47], [48], versus the small windows for simple stimuli [94] .

Finally, the superposition model is similar in many respects to a Bayesian model of bimodal integration. For example, in models developed by Ernst and colleagues [110], [111], maximal benefit due to bimodal discrimination occurs when the difficulty of each modality is roughly equated [112]. This is remarkably similar to the notion of physiological synchrony. Thus, Bayesian models could, presumably, be adapted to explain the reaction times and would also subsume the time window of integration concept. However, the advantage the superposition model has is that its neurophysiological implementation is immediately apparent. Bayesian models, in contrast, are usually more abstract, and it is unclear what their neural implementation would look like.

## Supporting Information

Figure S1 **Hit rate and False Alarm rate of one monkey.** **A:** Hit rate and false alarm rate from a single session. X-axes denotes bin number. Y-axes denotes percentage. **B:** Hit rate and false alarm rate from another session. Conventions as in A. **C:** Average hit rate and false alarm rate across all sessions for monkey 1. X-axes depict different types of metrics (Hit rate, False Alarm rate). Y-axes depict percentage. Error bars denote twice the standard error.
(PDF)

Figure S2 **Reaction time as a function of the inter stimulus interval for monkeys and humans.** **A**: Mean reaction times of monkey 1 as a function of the inter-stimulus interval for the three conditions of interest, auditory-only, visual-only and audiovisual for the +5 dB SNR condition. X-axes depict ISI in milliseconds. Y-axes depict reaction times in milliseconds. Error bars denote standard errors estimated using a bootstrap method. **B**: Mean gain in reaction times for monkey 1 for the audiovisual condition relative to the auditory-only condition as a function of the inter-stimulus interval for three SNRs (+22 dB, +5 dB, −10 dB). X-axes depict ISI in milliseconds. Y-axes depict the gain in reaction times in milliseconds. Error bars denote standard error of the mean estimated using a bootstrap method. **C**: Same analysis as A but for Monkey 2. **D**: Same analysis as B but for Monkey 2. **E**: Same analysis as A for human subjects. **F**: Same analysis as B for human subjects.
(PDF)

Figure S3 **Race models cannot explain audiovisual reaction times for monkeys.** **A**: Contour plot of the violation of race model as a function of both ISI and SNR for the reaction time data from Monkey 1. X-axes depict ISI in milliseconds. Y-axes depict SNR. Color bar denotes the amount of violation of the race model. **B**: Same analysis as A, but for monkey 2. Conventions are as in A.
(PDF)

Figure S4 **Proportional benefit in RT for the audiovisual condition compared to unisensory conditions.** **A**: Mean benefit in RT for the audiovisual condition expressed as a percentage of speedup relative to the minimum of mean visual-only and auditory-only RTs for monkey 1. X-axes depict SNR. Y-axes depict the benefit in percent. Error bars denote standard errors estimated through bootstrap. **B**: Same analysis as in A except for Monkey 2. Conventions as in A. **C:** Same analysis as in A except averaged across human subjects. Conventions as in A.
(PDF)

Figure S5 **Speeding up auditory RTs shifts the point of maximal integration.** **Left panels –** Simulated reaction times to visual, auditory and audiovisual conditions. X-axes depict SNR in dB. Y-axes the RT in milliseconds. From top to bottom, auditory-only RTs are sped up by 0, 40, 80 and 120 ms. **Right panels –** Benefit in simulated RT for the audiovisual compared to the auditory and visual-only conditions as a function of SNR for the scenarios shown in the left panel. X-axes depict SNR in dB. Y-axes the benefit in RT in milliseconds. One can see that the point of maximal integration and the shape of the benefit curve changes.
(PDF)

Figure S6 **Scenarios demonstrating the sensitivity of the principle of inverse effectiveness to stimulus characteristics.** **A, C, E –** Simulated reaction times to visual, auditory and audiovisual conditions. X-axes depict SNR in dB. Y-axes the RT in milliseconds. **B,D,F –** Benefit in simulated RT for the audiovisual compared to the auditory and visual-only conditions as a function of SNR for the scenarios shown in A,C,E. X-axes depict SNR in dB. Y-axes the benefit in RT in milliseconds. Note how in the first two scenarios (A,C and B, D) the simulated benefits follow the principle of inverse effectiveness. However for the last scenario (E,F), the simulated benefits do not follow it.
(PDF)

Text S1 **Effect of ISI on auditory, visual and audiovisual RTs.** A section describing how audiovisual integration in RTs are modulated by the inter-stimulus interval.
(PDF)
