# Supplementary figures and images for: Monkeys and Humans Share a Common Computation for Face/Voice Integration
Source: PLoS Comput Biol. 2011 Sep 29;7(9):e1002165. doi: 10.1371/journal.pcbi.1002165 (PMC3182859; doi:10.1371/journal.pcbi.1002165)

**A**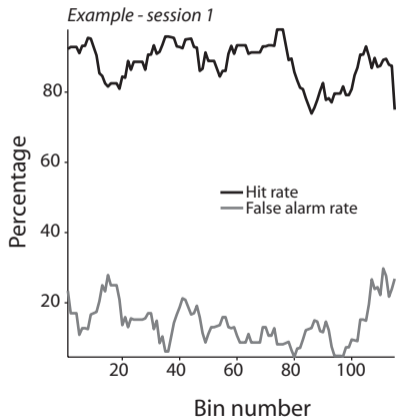**B**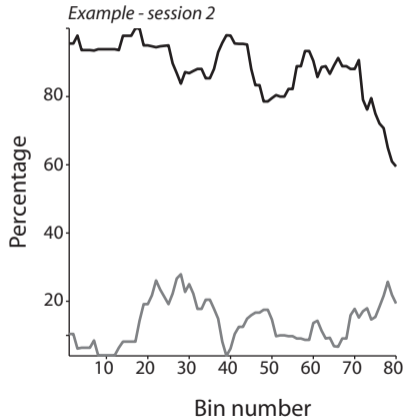**C**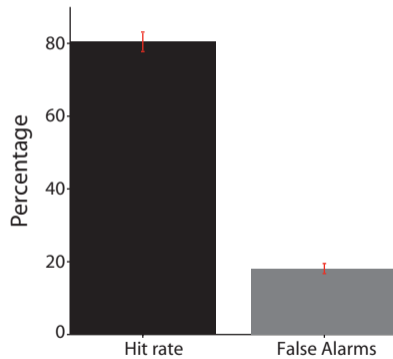

Supplement: Figure S1 — Hit rate and False Alarm rate of one monkey. A: Hit rate and false alarm rate from a single session. X-axes denotes bin number. Y-axes denotes percentage. B: Hit rate and false alarm rate from another session. Conventions as in A. C: Average hit rate and false alarm rate across all sessions for monkey 1. X-axes depict different types of metrics (Hit rate, False Alarm rate). Y-axes depict percentage. Error bars denote twice the standard error. (PDF) [file pcbi.1002165.s001.pdf]

**A**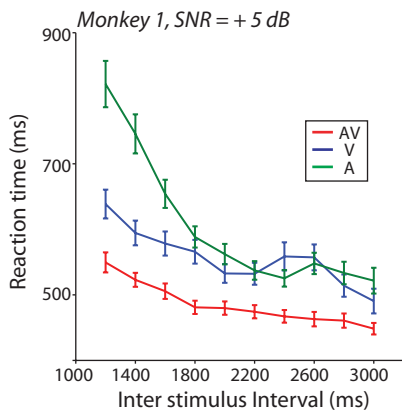**B**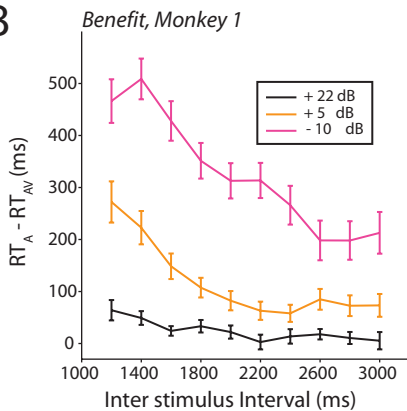**C**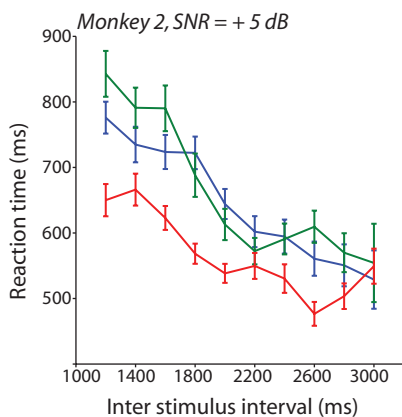**D**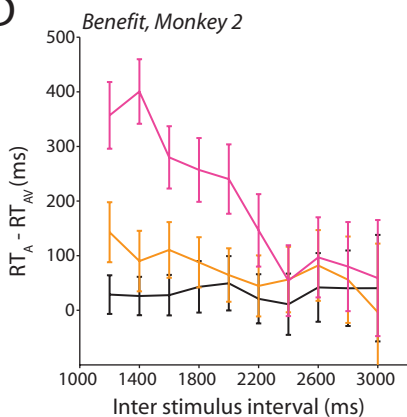**E**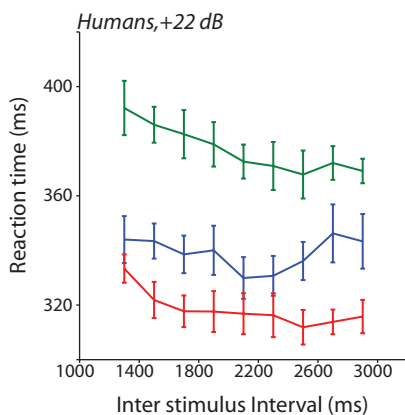**F**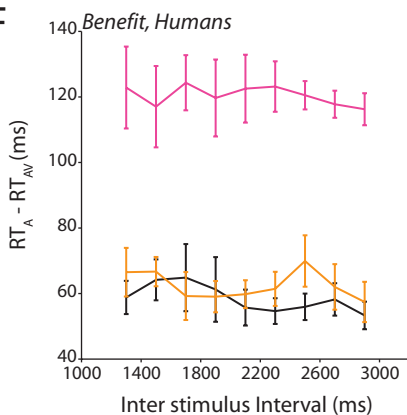

Supplement: Figure S2 — Reaction time as a function of the inter stimulus interval for monkeys and humans. A: Mean reaction times of monkey 1 as a function of the inter-stimulus interval for the three conditions of interest, auditory-only, visual-only and audiovisual for the +5 dB SNR condition. X-axes depict ISI in milliseconds. Y-axes depict reaction times in milliseconds. Error bars denote standard errors estimated using a bootstrap method. B: Mean gain in reaction times for monkey 1 for the audiovisual condition relative to the auditory-only condition as a function of the inter-stimulus interval for three SNRs (+22 dB, +5 dB, −10 dB). X-axes depict ISI in milliseconds. Y-axes depict the gain in reaction times in milliseconds. Error bars denote standard error of the mean estimated using a bootstrap method. C: Same analysis as A but for Monkey 2. D: Same analysis as B but for Monkey 2. E: Same analysis as A for human subjects. F: Same analysis as B for human subjects. (PDF) [file pcbi.1002165.s002.pdf]

**A***Monkey 1*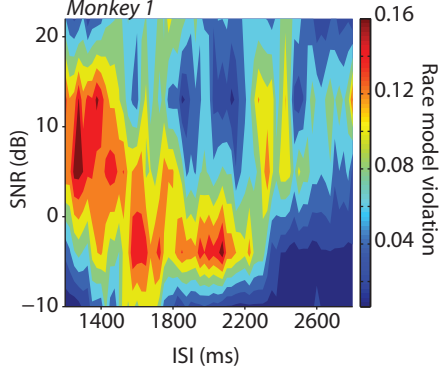**B***Monkey 2*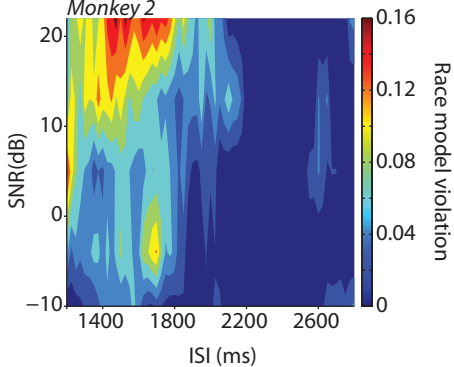

Supplement: Figure S3 — Race models cannot explain audiovisual reaction times for monkeys. A: Contour plot of the violation of race model as a function of both ISI and SNR for the reaction time data from Monkey 1. X-axes depict ISI in milliseconds. Y-axes depict SNR. Color bar denotes the amount of violation of the race model. B: Same analysis as A, but for monkey 2. Conventions are as in A. (PDF) [file pcbi.1002165.s003.pdf]

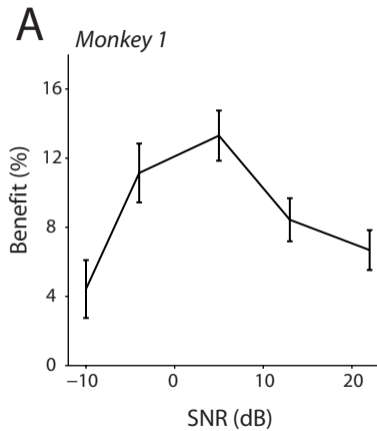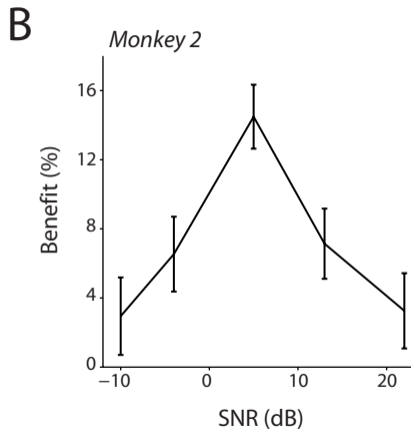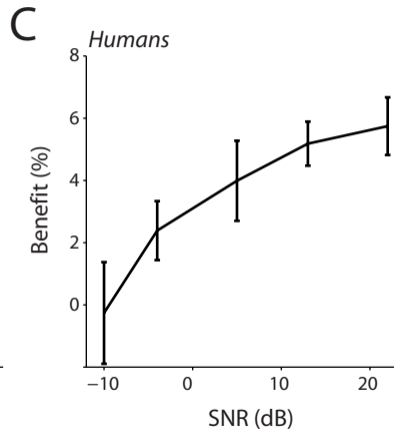

Supplement: Figure S4 — Proportional benefit in RT for the audiovisual condition compared to unisensory conditions. A: Mean benefit in RT for the audiovisual condition expressed as a percentage of speedup relative to the minimum of mean visual-only and auditory-only RTs for monkey 1. X-axes depict SNR. Y-axes depict the benefit in percent. Error bars denote standard errors estimated through bootstrap. B: Same analysis as in A except for Monkey 2. Conventions as in A. C: Same analysis as in A except averaged across human subjects. Conventions as in A. (PDF) [file pcbi.1002165.s004.pdf]

## RT profiles

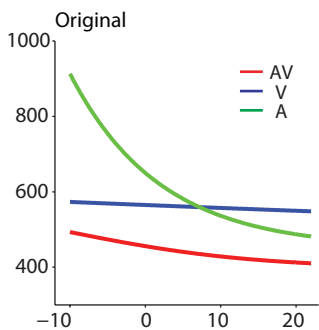

## Benefit profiles

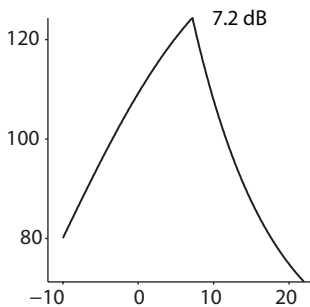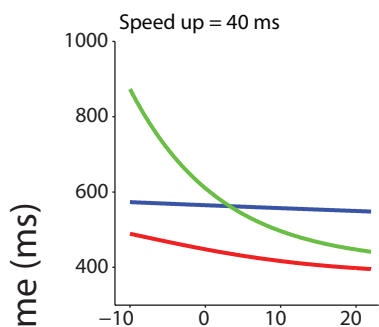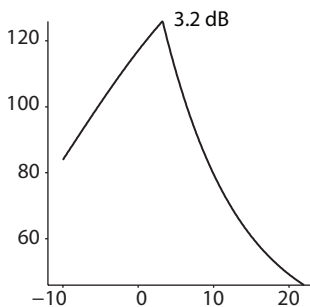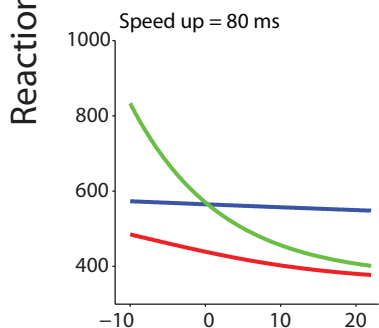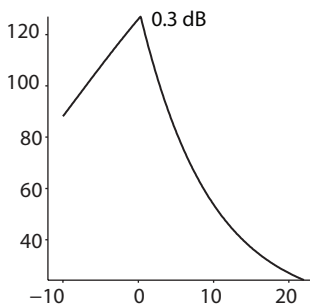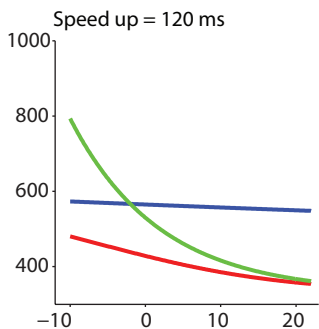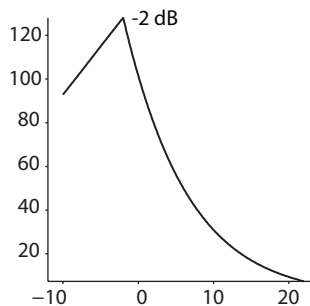

Benefit (ms)

Reaction time (ms)

SNR (dB)

Supplement: Figure S5 — Speeding up auditory RTs shifts the point of maximal integration. Left panels – Simulated reaction times to visual, auditory and audiovisual conditions. X-axes depict SNR in dB. Y-axes the RT in milliseconds. From top to bottom, auditory-only RTs are sped up by 0, 40, 80 and 120 ms. Right panels – Benefit in simulated RT for the audiovisual compared to the auditory and visual-only conditions as a function of SNR for the scenarios shown in the left panel. X-axes depict SNR in dB. Y-axes the benefit in RT in milliseconds. One can see that the point of maximal integration and the shape of the benefit curve changes. (PDF) [file pcbi.1002165.s005.pdf]

**A**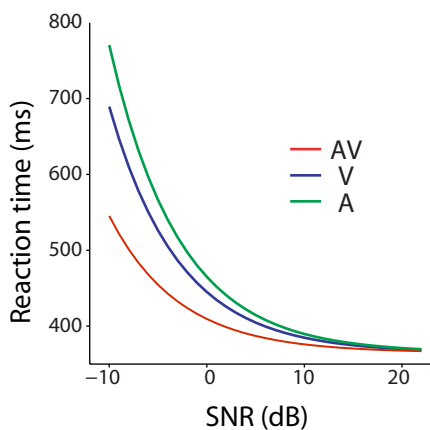**B**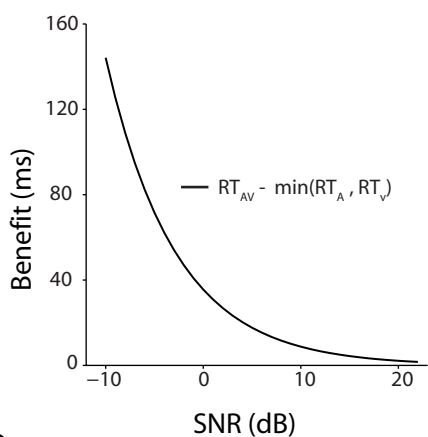**C**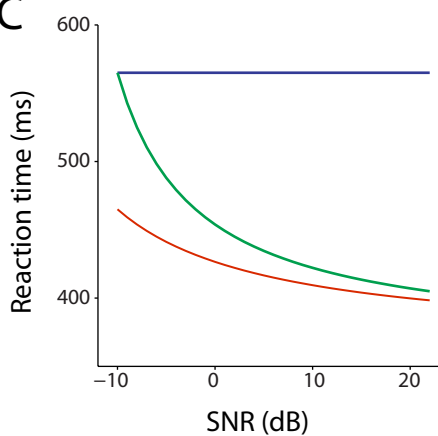**D**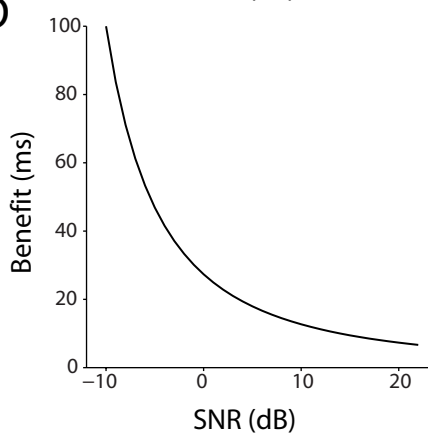**E**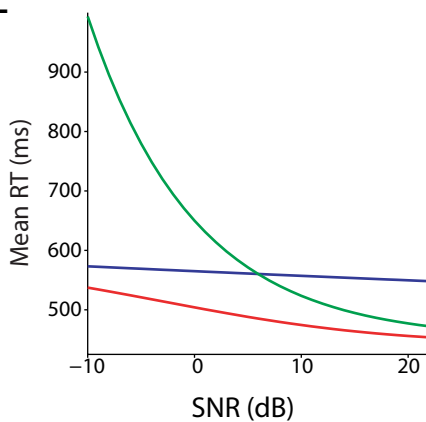**F**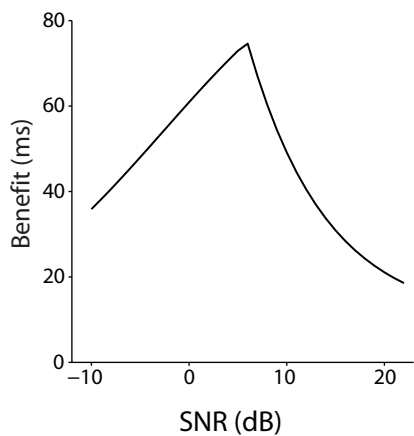

Supplement: Figure S6 — Scenarios demonstrating the sensitivity of the principle of inverse effectiveness to stimulus characteristics. A, C, E – Simulated reaction times to visual, auditory and audiovisual conditions. X-axes depict SNR in dB. Y-axes the RT in milliseconds. B,D,F – Benefit in simulated RT for the audiovisual compared to the auditory and visual-only conditions as a function of SNR for the scenarios shown in A,C,E. X-axes depict SNR in dB. Y-axes the benefit in RT in milliseconds. Note how in the first two scenarios (A,C and B, D) the simulated benefits follow the principle of inverse effectiveness. However for the last scenario (E,F), the simulated benefits do not follow it. (PDF) [file pcbi.1002165.s006.pdf]
