# Supplementary material for: Monkeys and Humans Share a Common Computation for Face/Voice Integration
Source: PLoS Comput Biol. 2011 Sep 29;7(9):e1002165. doi: 10.1371/journal.pcbi.1002165 (PMC3182859; doi:10.1371/journal.pcbi.1002165)
Supplement: Text S1 — Effect of ISI on auditory, visual and audiovisual RTs. A section describing how audiovisual integration in RTs are modulated by the inter-stimulus interval. (PDF) [file pcbi.1002165.s007.pdf]

Supplementary Text S1:

Integration benefits in monkeys but not humans seem to be modulated by the temporal uncertainty of the sensory stimuli

We have emphasized the dependence of the behavioral performance of monkeys and humans in relation to the properties of the sensory stimuli such as modality and SNR. However, other internal factors such as the expectation of sensory stimuli can also modulate RT and performance and thus change the points at which behavioral gain is present. In particular, keeping with classical redundant signal experimental designs[1], we chose a uniformly random inter-stimulus interval. However, these uniform inter-stimulus intervals can also bias the expectation of the animal. Subjects' expectations for the next stimulus increase as time from previous reward increases. We investigated whether this had any effect on the RT of the animals and humans and, if so, whether it modulated the reaction time gain for the audiovisual condition.

Figure S2A plots the RT of monkey 1, for the SNR of + 5 dB, as a function of the inter-stimulus interval (ISI). Each point corresponds to the average of all the RTs that were recorded when the ISI was within a 400-ms bin. The center of each bin was shifted by 200 ms. As ISI increases, the RT to all three conditions for this SNR decreases and is lowest when the ISI values were close to the end of the trained interval. To identify if the ISI had any effect on the multisensory gains in the monkeys, we computed the gain in the multisensory condition relative to the auditory-only condition for each of the ISI bins. Results of this analysis from monkey 1 are plotted in Figure S2B. As ISI increases, the gain in the multisensory condition relative to the auditory-only condition decreases and is smallest at the end of the ISI interval. Furthermore, these effects are also modulated by the difficulty of the auditory-only condition. For the lowest SNR there was a greater effect of the ISI on the multisensory gain than on the highest SNR. For example, the slope and 95% confidence

interval for linear regression analysis is -0.028(-0.041,-0.014) for the SNR of + 22 dB, -0.1(-0.16, -0.05) for the intermediate SNR of +5 dB and is -0.17(-0.22, -0.13) for the lowest SNR of -10 dB. Thus, as SNR decreases, the dependence of the multisensory benefit on the ISI increases. Very similar patterns are seen for Monkey 2 (Figures S2C, S2D). For humans, RTs for the visual-only and auditory-only conditions for the + 22 dB SNR condition do change with the expectation of the participant, but gains are not modulated as much or only weakly (Figure S2E). Figure S2F shows the gain for the audiovisual condition relative to the auditory-only condition for the six human subjects. The slope of these gain curves relative to the ISI is not significantly different from zero for the -10 and +5 dB SNRs ($p > 0.05$, regression test) and was significantly different from zero only for the +22 dB SNR. Therefore, temporal expectation modulates the multisensory gain strongly in monkeys but only weakly in humans—at least for the stimuli used here.

1. Miller J (1982) Divided attention: Evidence for coactivation with redundant signals. Cognitive Psychology 14: 247-279.
